# Steatosis and steatohepatitis found in adults after death due to non-burn trauma

**DOI:** 10.6061/clinics/2019/e1070

**Published:** 2019-10-09

**Authors:** Paulo Reis-Júnior, Ryan Tanigawa, Gustavo Heluani Antunes de Mesquita, Natalia Basan, Venâncio Alves, Luiz Augusto Carneiro D’Albuquerque, Wellington Andraus

**Affiliations:** Faculdade de Medicina, Universidade Federal do Tocantins, Palmas, TO, BR; Divisao de Patologia, Hospital das Clinicas HCFMUSP, Faculdade de Medicina, Universidade de Sao Paulo, Sao Paulo, SP, BR; Faculdade de Medicina FMUSP, Universidade de Sao Paulo, Sao Paulo, SP, BR; Departamento de Gastroenterologia, Hospital das Clinicas HCFMUSP, Faculdade de Medicina, Universidade de São Paulo, São Paulo, SP, BR; Servico de Transplante e Cirurgia do Figado, Hospital das Clinicas HCFMUSP, Faculdade de Medicina, Universidade de Sao Paulo, Sao Paulo, SP, BR

**Keywords:** Steatosis, Steatohepatitis, Fatty Liver, Liver Disease, Authopsy

## Abstract

**OBJECTIVE:**

With the increasing prevalence of steatosis, the number of steatotic liver grafts from deceased donors is also increasing. Thus, determining the prevalence and the population risk factors of steatosis may assist in risk stratification. The aim of this study was to evaluate the prevalence and predictors of steatosis and steatohepatitis among livers from adults who died due to non-burn trauma.

**METHODS:**

Specimens were collected from 224 adults undergoing autopsy at a regional autopsy referral center from September 2011 to April 2013. Histopathological examination was performed on six samples obtained from different lobes of each liver. The outcomes of interest were the presence of steatosis, steatohepatitis, NASH inflammation and NASH fibrosis. The main predictors were body mass index, abdominal circumference, liver weight and volume, presence of cholelithiasis, and siderosis. Our modeling strategy made use of a series of generalized linear models with a binomial family.

**RESULTS:**

Our sample had a mean age of 40 years; steatosis was diagnosed in 48.2% of cases, and steatohepatitis was diagnosed in 2.7%. The presence of a high proportion of fatty changes was more prevalent among males and older individuals, with the most affected age group being 41-60 years. When evaluating the crude odds ratio for steatosis, the factors significantly associated with an increased risk of steatosis were greater abdominal circumference, BMI, and liver weight and the presence of siderosis.

**CONCLUSION:**

Our study reinforces the role of older age, obesity and hepatomegaly as predictors of fatty liver disease. These variables should be considered in the assessment of fatty changes in the livers of potential liver donors.

## INTRODUCTION

Steatosis or the abnormal retention of triglycerides and other fat vacuoles in hepatocytes is a common cause of hepatic disease (1). It has a high prevalence among heavy alcohol drinkers (2), as well as those with diagnoses of hepatitis C infection (1), diabetes, and dyslipidemia and obese individuals (3). Non-alcoholic fatty liver disease (NAFLD) can occur concomitantly with hepatocyte inflammation, in which case it is referred to as non-alcoholic steatohepatitis (NASH) (1,3). With the increasing prevalence of steatosis, the number of steatotic liver grafts from deceased donors is also increasing (4). Because the transplantation of steatotic liver grafts is associated with complications including reperfusion injuries and graft failure (5), it is important to understand steatosis risk factors in an otherwise normal population. Routine liver biopsy as a pretransplantation work-up is challenging because only over 20% of all liver transplants in the United Network for Organ Sharing (UNOS) have a recorded liver donor biopsy (5). The lack of liver donor biopsy is further worsened in settings in which histopathological services are not provided on a 24-hour basis in the transplantation centers (6). Thus, determining the actual prevalence and the risk factors for steatosis in the normal population may assist in stratifying the risk of steatosis in potential liver donors soon after harvesting the liver.

The population prevalence of steatosis depends on the diagnostic test being used. Currently, modalities for diagnosing steatosis range from imaging techniques to biopsy, and these techniques have different accuracy levels in different contexts. Imaging techniques are generally used to screen for patients who may need biopsy, and these techniques also have a set of limitations. Ultrasound, for example, is an operator-dependent procedure with variable results (7) and is limited in terms of its accuracy with regard to detecting mild steatosis (8) as well as examining morbidly obese patients (9,10). Similarly, computerized tomography (CT) scanning can detect mild steatosis relatively less accurately, unless unsafe levels of radiation are used (11). Furthermore, magnetic resonance imaging (MRI) and magnetic resonance spectroscopy (MRS) have superior detection rates compared to those of CT and ultrasound but are also limited in their ability to produce information that can be used to stage NAFLD (12). Moreover, these tools are expensive and not necessarily available during the peri-harvesting procedure.

On the other hand, liver biopsy is considered the gold standard for diagnosis. This standard was established despite its shortcomings, including false negatives, in cases in which the distribution of non-alcoholic steatohepatitis lesions in the parenchyma is irregular (13,14) as well as high interobserver variability in evaluating lobular inflammation and ballooning (15). In addition, due to the invasiveness of this procedure, clinicians tend to not consider it a required step in the management of fatty liver disease, with diagnostic biopsy being performed only in select patients. Therefore, liver biopsy performed on live patients is usually not a practical option to determine the true prevalence among the general population.

Given the challenges of donor liver biopsy prior to transplantation and the limitations of contemporary diagnostic modalities in describing steatosis in the general population, evaluating cadaveric livers might provide a way to determine the risk of fatty liver disease, ultimately assisting clinicians in evaluating organ quality. To the best of our knowledge, however, no previous study has evaluated the predictors of steatosis and steatohepatitis in cadaveric livers. To bridge this gap in the literature, this study aimed to evaluate steatosis and steatohepatitis in the livers of adults after their deaths due to non-burn trauma.

## METHODS

### Study design

This was a prospective cohort study evaluating steatosis and steatohepatitis in adults who died due to non-burn trauma. The study is described in accordance with the STrengthening the Reporting of OBservational studies in Epidemiology (STROBE) guidelines (16). A total of 224 cases were included in this analysis.

### Ethics

Our study was approved by the Institutional Review Boards of the Schools of Medicine at the University of São Paulo (CAPPesq, HCFMUSP) and the Federal University of Tocantins (UFT) in accordance with the general direction of the Legal Medical Institute (IML) of Palmas-TO. Informed consent was obtained from all family members or legal guardians of the investigated deceased and was signed prior to any study protocol being implemented.

### Setting

Specimens were collected from 224 adult cadavers undergoing autopsy at the Legal Medical Institute (IML) of Palmas, Brazil, from September 2011 to April 2013. The Legal Medical Institute (IML) is located in Northern Brazil, serving a population of approximately 320,000 people living in 17 surrounding towns. All autopsies were part of a forensic investigation performed on those with unnatural deaths, with mechanisms including accidents and other non-burn trauma.

### Participants

**Figure 1:**
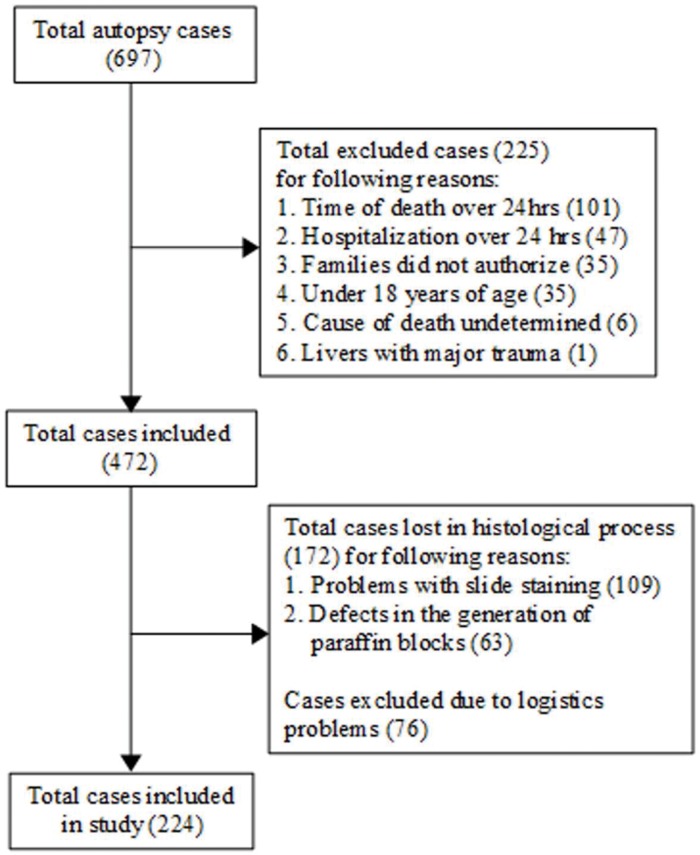
Flowchart detailing the inclusion and exclusion of cases.

We only included individuals who were 18 years or older and died due to a non-burn trauma. We excluded those who (1) had died more than 24 hours prior to autopsy to provide adequate time for analysis, without the risk of significant histological changes, (2) had spent more than 24 hours in the hospital to avoid histological changes due to therapeutic action and early hepatic dysfunction during hospitalization (17), (3) had experienced major direct trauma to the liver, and (4) had an undetermined cause of death; we also excluded those whose families did not authorize the procedure. Cases presenting technical challenges were also excluded, including problems with slide staining, failure to acquire sample tissue, logistical problems and defects in the generation of paraffin blocks (Figure 1).

### Sample examination

After obtaining a history of alcohol consumption and any previous liver disease from a first-degree relative, the anthropometric parameters of each corpse were measured, and samples were obtained for pathological examination. The histopathological examination was conducted by a pathologist in the Research Laboratory on Liver Pathology at the University of São Paulo. From a sagittally sectioned liver, a total of six samples, each measuring 2 centimeters by 1.5 centimeters, were obtained from the subcapsular and intraparenchymal areas of the right posterior, central and left lateral lobes of the liver. A total of 1,344 biopsies were therefore collected from the 224 deceased individuals. All samples were processed, sectioned, and stained with hematoxylin-eosin, Perls, trichrome Masson and Picrosirius; a total of 5,376 slides were examined for the purposes of this study.

### Outcomes

The outcomes of interest were the detection of steatosis, steatohepatitis, NASH inflammation and NASH fibrosis on the histopathological examination. Steatosis was determined by estimating the proportion of hepatocytes containing fat droplets (macrovesicular or microvesicular), which was converted to grades as follows: Grade 0 (less than 5%), Grade 1 (5-33%), Grade 2 (33-66%) and Grade 3 (above 66%). The topography of steatosis cells was categorized as zone 1 (periportal area), zone 3 (centrilobular area) and zone 2 (intermediate between zones 1 and 3). Steatohepatitis was determined by the NAFLD activity score (NAS), as proposed by the Non-alcoholic Steatohepatitis Clinical Research Network (NASH-CRN) Pathology Committee, which is based on the sum of each histological component evaluated as follows: steatosis (0-3), ballooning (0-2), and lobular inflammation (0-3). Cases with NAS scores of 0-2 were considered to not have steatohepatitis, those with scores of 3-4 were considered to have borderline steatohepatitis, and those with scores greater or equal to 5 were considered to have a definitive steatohepatitis diagnosis (18). The histological lesions considered while determining the NASH grade included steatosis, ballooning and inflammation. These were graded semiquantitatively on a scale where 0 = absent, 1 = mild, 2 = moderate, and 3 = severe (18). NASH inflammation was assessed through an evaluation of inflammatory foci per 20X field characterized by a mixed inflammatory infiltrate composed of neutrophils and mononuclear cells, evaluated as Grade 0 (no foci), Grade 1 (less than 2 foci), Grade 2 (2-4 foci) and Grade 3 (greater than 4 foci) (18). NASH fibrosis pathological staging was performed as proposed by Kleiner: 1 = perisinusoidal or periportal; 2 = perisinusoidal and periportal; 3 = bridging fibrosis and 4 = cirrhosis (18).

### Predictors

Our main predictors were body mass index (weight in kilograms over the square of height in meters); abdominal circumference in centimeters (cm) at the umbilical level; liver weight in grams; liver volume calculated as a product of the height (cm), width (cm) and length (cm) of the liver; the presence of cholelithiasis; hepatic iron overload (siderosis) assessed based on the cellular distribution of iron deposits within the Kupffer cells, hepatocytes, and macrophages; and the presence of siderosis, histologically graded as 0 = granules absent or barely discernible, 1 = granules barely discernible but present, 2 = discrete granules resolved at x 100, 3 = 3 discrete granules resolved at x 50 or 4 = granules visible to the naked eye (19). Any siderosis was considered as the presence of siderosis among Kupffer cells, hepatocytes or macrophages.

### Potential confounding variables

Our potential confounders were selected based on evidence from previous studies combined with clinical judgment (20). Specifically, we selected age and sex as potential confounding variables.

### Data analysis

Our exploratory analysis started by evaluating the distributions, frequencies and percentages for each of the numeric and categorical variables. Categorical variables were evaluated for near-zero variation (21). Extensive graphical displays were used for both univariate analysis and bivariate associations, accompanied by broader tests such as the Maximal Information Coefficient (22) and Nonnegative Matrix Factorization (23) algorithms for numeric variables.

Our modeling strategy made use of a series of generalized linear models with a binomial family to assess the associations of the investigated variables with steatosis or steatohepatitis as the outcome variables. The predictors included body mass index (body weight in kilograms over height squared in meters), abdominal circumference (centimeters), liver weight (grams), liver volume (cubic centimeter), the presence of cholelithiasis, and hepatic iron overload (siderosis).

We then performed an analysis using unsupervised tree models for hierarchical clustering (24) to identify the most common associations and hierarchical patterns among the following variables: abdominal circumference, liver weight, liver volume, the presence of cholelithiasis, and hepatic iron overload (siderosis).

All analyses were performed using the R language and the following packages: ggplot2 and rmarkdown.

## RESULTS

### Participant characteristics

A total of 697 cases were assessed initially, with 225 excluded either because the time of death was more than 24 hours prior to autopsy, the hospitalization duration was over 24 hours, the liver was severely damaged by trauma, the cause of death was undetermined, the individual was younger than 18 years of age or the family did not provide consent. A further 248 were lost in the histological process due to problems with slide staining and the failure to acquire samples or logistics. Thus, 224 cases were included in our final analysis. Our sample had a mean age of 39.7 (±16.57) years, with a higher proportion of fatty liver changes observed in males, and the most commonly affected age group was the group from 41 to 60 years old. Steatosis was diagnosed in 48.2% of all cases, with steatohepatitis being detected in 2.7% of the cases (Table 1).

### Outcomes

Among those with hepatocyte fatty changes, a majority of cases (80.6%) were Grade 1 steatosis, with 50% of steatohepatitis cases classified as Grade 2. Ballooning was detected in 15/224 (6.7%) cases, 60% of which were Grade 1. Less than half (**41.5%**) of the cases were classified as Grade **1 NASH** inflammation. Four cases were NASH fibrosis, and three were classified as steatosis. Steatohepatitis was identified in two of these steatosis cases. Hepatic iron overload was detected in 8% of all cases and 13% of cases with steatosis, and it was present in hepatocytes, Kupffer cells or macrophages. However, iron overload was only present in one case of steatohepatitis. Although the presence of Mallory Denk bodies is not regarded as a characteristic feature of steatohepatitis, it was present in 5 out of 6 cases of steatohepatitis (Table 2).

The odds of steatosis (unadjusted and adjusted) were higher in individuals with increased abdominal circumference, BMI, and liver weight. In addition, the presence of any siderosis was also associated with steatosis. These associations were still significant after adjusting for age and sex (Table 3).

**Figure 2:**
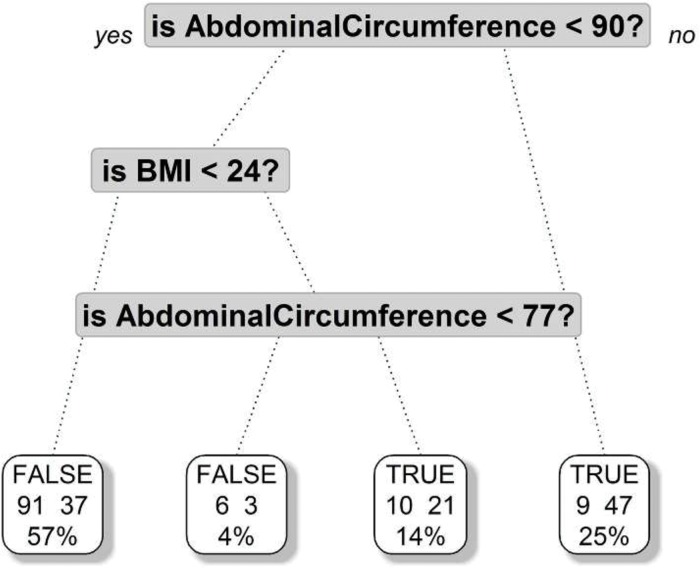
Tree regression model with risk factors for steatosis.

A further tree regression model demonstrated that an abdominal circumference greater than 90 centimeters was the most important risk factor for steatosis, and a BMI greater than 24 was a risk factor in those with abdominal circumferences greater than 77 centimeters (Figure 2).

## DISCUSSION

To the best of our knowledge, this is the first study evaluating steatosis and steatohepatitis in adults who died due to non-burn trauma. We found a 48.2% prevalence of steatosis, with a significant upward trend in higher age groups, except in individuals over 60 years. An increased risk of steatosis was associated with obesity and hepatomegaly, as evaluated through the measures of BMI, abdominal circumference, liver weight and volume, and siderosis. We also found a total of six cases of steatohepatitis and four cases of NASH fibrosis, two of which were in individuals with steatohepatitis.

Our study found that fatty liver disease is common among individuals who have died due to non-burn trauma. Importantly, the overall rate of fatty liver disease in our sample was higher than in most previous population-based studies. A systematic review reported the prevalence of NAFLD in the general population to be as high as 35% (25-28). However, most previous prevalence reports were based on noninvasive diagnostic protocols, such as radiological modalities or serum biomarkers, which tend to underestimate the prevalence of this condition. It is also noteworthy that autopsy series describing the prevalence of NAFLD have presented wide variability in their results, ranging from 3 to 50% (28-30). There are a number of possible explanations for this variation, including heterogeneity in sample characteristics regarding age, ethnicity, health status, history of alcohol consumption, and dietary habits (31,32). Moreover, several methodological factors can affect the histopathological evaluation, including the interval between the time of death and the examination, as well as the quality of biopsy techniques. Finally, histological findings and diagnostic categories are subject to interobserver disagreement (33). These findings emphasize the importance of standardizing protocols in future studies evaluating fatty liver disease among potential organ donors considered to be in good health, so that results can be deemed comparable.

The pathogenesis and natural history of NAFLD have been the subjects of controversy, as multiple theories have been proposed. Several factors may be involved, including insulin resistance, oxidative stress, inflammation, and dysfunctional hepatocyte apoptosis (34-36). The role of oxidative stress in the development of NAFLD may explain the positive association between siderosis and steatosis in our sample, as hepatic iron overload increases oxidative damage to tissues (37-40). It is possible that biomarkers of iron metabolism and imaging techniques evaluating hepatic iron overload may have predictive value in fatty liver disease.

In our study, only a small percentage of subjects had steatohepatitis and/or NASH fibrosis. Although theoretical models have proposed that NAFLD comprises a continuum of hepatocyte lesions from steatosis to NASH and fibrosis (35,41-43), these models have been questioned in the most recent literature. For example, several studies have argued that steatosis and NASH are distinct conditions within the NAFLD concept, as both exhibit different pathophysiological, histological, and clinical features (44,45). Further studies should provide a more comprehensive model explaining the underlying fatty liver disease mechanisms, including the relationship between steatosis and NASH, as well as variables associated with prognosis in both conditions.

The higher risk of steatosis and NASH among obese individuals in our sample is consistent with the literature emphasizing the importance of obesity as a risk factor for fatty liver disease. Accordingly, NAFLD is considered the hepatic component of metabolic syndrome, a cluster of conditions including central fat accumulation, insulin resistance, diabetes, dyslipidemia, and hypertension (46-51). The deposition of fat in the liver may lead to hepatomegaly, which is consistent with the higher risk of NAFLD among subjects with increased liver weight and volume (52,53). Previous studies estimated the rates of NAFLD among obese individuals to be as high as 74% (54). The association between obesity and NAFLD is likely a consequence of the large sources of fatty acids in obese individuals, which originate from adipose tissue and are combined with the less effective insulin-mediated suppression of lipolysis (55,56). The exact extent of the contribution of obesity as a cause of NAFLD is difficult to determine, as obese individuals also present high rates of other risk factors for NAFLD, such as dyslipidemia and diabetes.

Another finding in our sample was the significantly higher prevalence of fatty liver disease and especially of steatohepatitis among individuals who were older, possibly as a result of higher rates of metabolic disorders in this age group (57,58). These individuals will also typically have a longer disease duration. Considering theories that describe NAFLD as a continuum, higher rates of NASH among older subjects might indicate disease progression (36,59,60). Conversely, in our sample, this trend did not extend to individuals over 60 years. The relatively small number of subjects in this age range may have contributed to our findings because the progressively higher risk of NAFLD among older individuals has been consistently reported in a number of previous studies (61,62).

Despite filling a gap in the literature, our study does have limitations. First, a significant number of samples were lost due to technical issues. In spite of this problem, data from a large number of individuals were still obtained. Second, because we used a convenience sample, the external validity of our findings can be questioned. Despite this limitation, our sample was obtained from a referral center in charge of conducting autopsies for the entire region, which might have minimized our selection bias. Third, we did not account for trauma-related oxidative stress, which might have artificially inflated the rates of steatosis when compared with other causes of death (25,26). Our analysis, however, included data from a large sample of individuals, providing robust results regarding the factors predicting NAFLD among potential liver donors in similar populations. Although the impact of oxidative stress is still controversial, a control group of individuals who died from nontraumatic causes could be used to adjust the rate of steatosis in future studies. Fourth, we did not consider the roles of other comorbidities, such as alcohol abuse, hepatitis, and diabetes, as predictors of steatosis or steatohepatitis. Nevertheless, these conditions have already been recognized as risk factors for steatosis in a number of previous studies (1-3,25-27). Finally, the histopathological findings in our biopsy samples were not validated through agreement across different pathology specialists.

In conclusion, our study reinforces the roles of older age, obesity and hepatomegaly as predictors of fatty liver disease. These variables should be considered in the assessment of liver fatty changes among potential liver donors. Further studies should focus on the development of reliable algorithms for diagnosing fatty liver disease, which may include clinical aspects, laboratory findings, and imaging tests. Finally, larger, longitudinal studies should expand the knowledge of the epidemiological and pathophysiological features of fatty liver disease, providing support for future clinical guidelines.

## AUTHOR CONTRIBUTIONS

Reis-Júnior P, Alves V, D’Albuquerque LAC and Andraus W contributed to the conception and design of the study. Reis-Júnior P, Tanigawa R, Alves V and Andraus W contributed to the analysis and interpretation of the data. Reis-Júnior P and Basan N contributed to data collection. Reis-Júnior P, Tanigawa R, Mesquita GHA contributed to the drafting of the manuscript. Alves V, Mesquita GHA and Andraus W contributed to the review of the manuscript. Reis-Júnior P, Alves V and Andraus W approved the final version of the manuscript.

## Figures and Tables

**Table 1:** Sample characteristics with anthropometric data stratified by steatosis and steatohepatitis.

Variable	Total (224)	Steatosis (108)	Steatohepatitis (6)
Age	39.7 (±16.57)	41.75 (±15.02)	47.17 (±8.95)
Age Categories (years)			
- less than or equal to 20	24 (10.7%)	7 (6.5%)	0 (0%)
- 21 to 40	107 (47.8%)	45 (41.7%)	1 (16.7%)
- 41 to 60	69 (30.8%)	46 (42.6%)	5 (83.3%)
- greater than 60	24 (10.7%)	10 (9.3%)	0 (0%)
Female	41 (18.3%)	19 (17.6%)	0 (0%)
BMI	24.25 (±4.69)	26.04 (±4.99)	31.52 (±5)
Liver Volume (cm^3^)	3403.67 (±1771.02)	3624.85 (± 1889.62)	6148 (±2840.39)
Liver Weight (g)	1586.41 (±412.54)	1674.59 (±469.02)	2228.17 (±791.03)
Abdominal Circumference (cm)	84.65 (±18.05)	91.59 (±19.56)	113.33 (±21.56)

**Table 2:** Histopathological data from study sample, stratified by steatosis and steatohepatitis.

Variable [Missing]	Total (224)	Steatosis (108)	Steatohepatitis (6)
Steatosis Grade [116]			
-1	87 (80.6%)	87 (80.4%)	2 (33.3%)
-2	11 (10.2%)	11 (10.3%)	3 (50%)
-3	10 (9.3%)	10 (9.3%)	1 (16.7%)
Ballooning Grade [209]			
-1	9 (60%)	9 (60%)	2 (33.3%)
-2	3 (20%)	3 (20%)	2 (33.3%)
-3	3 (20%)	3 (20%)	2 (33.3%)
NASH Inflammation Grade [0]			
0	129 (57.6%)	14 (13%)	0 (0%)
-1	93 (41.5%)	92 (85.2%)	4 (66.7%)
-2	2 (0.9%)	2 (1.9%)	2 (33.3%)
NASH Inflammation [0]	95 (42.4%)	94 (87%)	6 (100%)
Mallory Denk Bodies [0]	8 (3.6%)	8 (7.5%)	5 (83.3%)
NASH Fibrosis Grade [0]			
0	220 (98.2%)	105 (97.2%)	4 (66.7%)
-1	2 (0.9%)	1 (0.9%)	0 (0%)
-2	2 (0.9%)	2 (1.9%)	2 (33.3%)
NASH Fibrosis [0]	4 (1.8%)	3 (2.8%)	2 (33.3%)
Siderosis Kupffer Cells Grade [0]			
0	214 (95.5%)	100 (92.6%)	5 (83.3%)
-1	9 (4%)	7 (6.5%)	1 (16.7%)
-2	1 (0.4%)	1 (0.9%)	0 (0%)
Siderosis Kupffer cells [0]	10 (4.5%)	8 (7.4%)	1 (16.7%)
Siderosis Hepatocytes Grade [0]			
0	220 (98.2%)	106 (98.1%)	6 (100%)
-1	4 (1.8%)	2 (1.9%)	0 (0%)
Siderosis Hepatocytes [0]	4 (1.8%)	2 (1.9%)	0 (0%)
Siderosis Macrophage [0]			
0	219 (97.8%)	103 (95.4%)	6 (100%)
-1	3 (1.3%)	3 (2.8%)	0 (0%)
-3	2 (0.9%)	2 (1.9%)	0 (0%)
Siderosis Macrophages	5 (2.2%)	5 (4.6%)	0 (0%)
Any Siderosis [1]	18 (8%)	14 (13%)	1 (16.7%)
Cholelithiasis [0]	3 (1.3%)	2 (1.9%)	0 (0%)

**Table 3:** Crude odds ratio (unadjusted and adjusted) and 95% Confidence Intervals of Steatosis diagnosis.

Variables	Steatosis Unadjusted OR (CI 95%)	Steatosis Adjusted OR (CI 95%)
Abdominal Circumference	3.72 (2.13, 6.48)	3.51 (1.95, 6.32)
BMI	3.25 (1.88, 5.61)	3.25 (1.82, 5.82)
Liver Volume	1.49 (0.88, 2.52)	1.47 (0.85, 2.56)
Liver Weight	1.92 (1.13, 3.27)	1.86 (1.05, 3.29)
Siderosis Hepatocytes	1.08 (0.15, 7.77)	1.09 (0.14, 8.37)
Siderosis Kupffer cells	4.56 (0.95, 21.98)	3.75 (0.74, 19.08)
Any Siderosis	4.17 (1.33, 13.1)	4.12 (1.27, 13.35)
